# Intention to use vasectomy and associated factors among married men in Addis Ababa, Ethiopia

**DOI:** 10.1186/s12889-020-09316-x

**Published:** 2020-08-12

**Authors:** Jemila Nesro, Endalew Gemechu Sendo, Nete Tofik Yesuf, Yitagesu Sintayehu

**Affiliations:** 1 Department of Midwifery, Jimma University, Jimma, Ethiopia; 2 Department of Nursing and Midwifery, College of Health Sciences, Addis Ababa University, P.O. Box 1176, Addis Ababa, Ethiopia; 3 Department of Midwifery, College of Health and Medical Sciences, Haramaya University, P.O. Box 235, Harar, Ethiopia

**Keywords:** Family planning, Vasectomy, Knowledge, Attitude, Intention, Addis Ababa

## Abstract

**Background:**

Vasectomy is one of the highly effective and non-reversible types of long-term family planning methods for men. Ethiopia has a limited number of studies on the use of vasectomy, and they are focused on men rather than married men. The current study was aimed to identify the intention to use vasectomy as a method of contraception among married men in the study setting.

**Methods:**

A community-based cross-sectional study was conducted from February 1 – April 30, 2018. A sample of 422 married men was recruited using a systematic random sampling method. We conducted face to face interviews with a structured questionnaire (i.e. closed-ended questions). Data were entered into Epi data version 3.1 and SPSS version 23 used for data analysis. The statistical association between the outcome variable (Intention to use vasectomy) and the explanatory variables were first tested with binary logistic regression. Multivariable logistic regression was used to control for confounding effect of each predictor.

**Results:**

The study findings showed that the intention to use vasectomy as a method of family planning was reported as high (24%). About 34.8% of the respondents had good knowledge and nearly a quarter (23.2%) of them had a positive attitude toward the acceptance of vasectomy use. In multivariate analysis, age range between 30 and 39 years [AOR = 2.4, 95% CI = (1.16–4.82)], having good knowledge about vasectomy use [AOR = 6.22, 95% CI = (3.17–12.21)], and having a positive attitude toward vasectomy use [AOR = 7.81, 95% CI = (4.25–14.38)] were factors significantly associated to use vasectomy as compared to their counterparts.

**Conclusion:**

The level of acceptance of vasectomy (24%) was high compared to the level of its use in developing countries (i.e. if acceptability translates to use). The study revealed that age, good knowledge, and a positive attitude towards the use of vasectomy were important predictors of the intention to accept vasectomy. To further promote the use of vasectomy effective communication strategies in family planning programs are needed.

## Background

Ethiopia is the second-largest populated country next to Nigeria in Africa with an estimated population of 104.96 million [1]. This high number of people might result in a decrease in Growth Domestic Product (GDP) and increased pressure on resource distribution [1]. Ethiopia is one of the East African Countries with a high number of maternal mortality whereby 412 deaths are estimated to occur in every 100,000 live births [1, 2]. Among the top ten countries with high maternal mortality that shares 58% of the global maternal deaths, Ethiopia is the fourth in rank that is accountable for 4% of global maternal deaths yearly [1].

Family planning (FP) including vasectomy has positive effects in terms of viable socio-economic development and in reducing maternal deaths [3]. Contraception has clear health benefits since the prevention of unintended pregnancies results in a subsequent decrease in maternal morbidity and mortality [4]. Regrettably, most family planning programs in Ethiopia have mostly targeted women, and men often do not take part in reproductive health matters [5]. FP programs of the country have focused predominantly on women to space and/or limit child-bearing to reduce maternal and infant mortality. According to the current Ethiopian Demographic Health Survey (EDHS) report, the total fertility rate (TFR) was reported to be 4.6 children per woman and the unmet need for FP remains high at 22% in 2016 [2].

The most commonly used contraceptive method to regulate fertility for currently married women in Ethiopia is injectable (23%), followed by implants (8%). Currently, married women with 1–2 living children are more likely to use a modern contraceptive method than women with more than 5 children (42 and 28%), respectively [2]. Long-acting and permanent methods (LAPMs) of contraception (implants, intrauterine devices, tubal ligations (TLs), and vasectomies) are generally inaccessible due to their need for highly skilled providers and specialized equipment. LAPMs thus constitute < 5% of contraceptive methods used in Ethiopia [6]. For instance, according to EDHS 2011 report, only 3% of them had used long-acting and permanent methods; Intrauterine device (IUD), female sterilization, and implant were used by 0.2, 1.3, and 2.4% of women, respectively [7]. According to the Ethiopian Demographic Health Survey 2016 report, the rate of vasectomy use among married men is very low in Ethiopia (< 1%) [2].

Studies suggest that male involvement can increase uptake and continuation of family planning methods through improving spousal communication through pathways of increased knowledge or decreased male opposition. However, regardless of increasing evidence on the benefits of engaging men in reproductive health decision-making, fertility rates and unmet need for family planning remain high in many Sub-Saharan African countries [8], including Ethiopia. For decades, calls have been made to surge the participation of men in matters of reproductive health and family planning to reduce maternal mortality and morbidity [8]. One way to foster male involvement in family planning is to provide couples more contraceptive choices through the promotion of male-oriented methods including vasectomy. Vasectomy is a safe, simple, and effective method that is comparatively underused throughout the world. Although sterilization is the most widely used contraceptive method worldwide, tubal ligation accounts for more than five times as many procedures as vasectomy [9–12].

Vasectomy is a surgical method used in men to cut or tie the vas deferens. The vas is a tube that delivers sperm from the testicles. The purpose of vasectomy is to provide permanent birth control for men who do not want more children [13]. It is a permanent method of family planning, which is quite acceptable in many developed countries of the world [13, 14]. However, in most African countries including Ethiopia, there are still prevailing barriers to its acceptance by married men [12]. Worldwide 19% of women in combination are sterilized (through tubal ligation) versus 2.4% men by vasectomy [9]. The highest rates of vasectomy in Africa are reported in South Africa (0.7%) and Namibia (0.4%) but this is still much lower than the global average [10]. Likewise, the rate of men’s participation in the overall Family Planning program in Ethiopia is still low (0.1%), with less than 1% of married men used vasectomy [2]. While several factors might affect male’s uptake of vasectomy as a modern method of family planning, to the best of the researchers’ knowledge, Ethiopia has a limited number of studies on the use of vasectomy, and they are focused on men rather than married couples. The current study was aimed to identify the intention to use vasectomy as a method of contraception among married men in the study setting.

## Methods

### Study setting and period

A community-based cross-sectional study was conducted from February–April 2018 in the Gulele Sub-city of Addis Ababa. The Addis Ababa City Government has 10 sub-cities, of which Gulele sub-city was randomly selected for this study. Each sub-city has an average of 10–12 districts.

### Population and the eligibility criteria

All married men living in the Gulale sub-city were the source population while randomly selected married men living with their wives in the selected Districts were the sample population. All married men consented to participate in the study during the data collection period were eligible for the study. Excluded from the study were men who lived less than six months in the study area and not willing to be involved in the study.

### Sample size determination and sampling procedure

The sample size was determined by a single population proportion formula using a 50% prevalence and 5% margin error with a 95% confidence level.

$$ n=\frac{{\left(\mathrm{Z}\ \mathrm{a}/2\right)}^2\ \mathrm{p}\ \left(1\hbox{-} \mathrm{p}\right)}{d^2}=384+38\left(10\%\mathrm{nonresponse}\ \mathrm{rate}\right)=422. $$

Gulale sub-city has 10 districts, of which three Districts namely: Wereda three, Wereda seven, and Wereda eight were selected by simple random sampling (lottery method) for the study. Then, the calculated sample size of 422 was proportionately allocated for each wereda based on the number of households. A systematic random sampling technique was then used to select the study participants. Data were collected by face to face interview using a pretested structured questionnaire (i.e. closed-ended questionnaire).

### Measurement

Information about vasectomy was assessed by a 10-item knowledge survey adjusted from past studies [15, 16]. The scale for evaluating knowledge was from 0 to 10 scores. Right answers were offered a score of 1 and incorrect responses 0. The individuals who scored not exactly the mean estimation of respondents’ scores were considered to have poor knowledge while the individuals who scored greater than or equivalent to the mean estimation of respondents’ scores were considered as having good knowledge.

**Intention to use:** refers to potential men who answered “yes” for the inquiry “will you acknowledge vasectomy as a technique for FP later on?” Information about the meaning of vasectomy was quickly given to respondents that had never caught wind of it they could show whether they expected to utilize it or not.

**Attitude towards vasectomy as a method of FP:** A 10-item Likert scale with 5 response alternatives were adjusted from earlier studies [10, 17]. The response alternatives include ‘Strongly Disagree = 1, Disagree = 2, Neutral = 3, Agree = 4, and Strongly Agree = 5.’ The total score were computed for every respondent and it ranges from 10 to 50. The individuals who scored not exactly the mean worth were considered to have a negative attitude while the individuals who scored greater than or equivalent to the mean worth were considered as having a positive attitude.

### Data analysis

Data were entered using Epi data version 3.1 and SPSS version 23 used for data analysis. The statistical association between the outcome variable (Intention to use vasectomy) and the explanatory variables were first tested with binary logistic regression. Variables that showed statistical significance at the 0.05 level were put into the final model (multivariate analysis) to control for confounding variables. A 95% confidence interval (CI) was used and a Statistical significance was declared at *P* < 0.05. The inclusion of variables in the final model considered issues of multicollinearity.

### Ethics approval and consent to participate

Ethical clearance to conduct this research was sought from the Research and Ethical Review Committee of the School of Nursing and Midwifery, College of Health Sciences, Addis Ababa University. Participants of the study were informed about the objective of the study and asked to provide informed voluntary written consent. Confidentiality and anonymity were ensured. Anonymity was assured about the identity and other personal information of all study participants. Confidentiality and privacy of participants were maintained throughout the research process and the dataset has been kept in a locked cupboard and not accessible by any other person except the research team.

## Results

### Socio-demographic and reproductive health characteristics

A sample of 422 married men was involved in the study. The mean and standard deviation age of respondents was 38.23 (SD ±9.50) years. About 33.6% of them completed tenth grade whereas regarding their religion more than half (53.3%) were Orthodox Christian. About 2/3th (62.2%) of the respondents were employed [Table 1].

**Table 1:** Demographic and Socio-Economic characteristics of the study participants live in Gulele sub-city, Addis Ababa, Ethiopia [*n* = 422]

Variables		Frequency	Percentage
Age	20–29	95	22.5
	30–39	155	36.7
	40–49	123	29.1
	50–59	33	7.8
	60–69	16	3.8
Ethnicity	Amhara	122	28.9
	Oromo	134	31.8
	Tigray	58	13.7
	Gurage	59	14
	South	40	9.5
	Others	9	2.1
Educational level	Grade ten and below	142	33.6
	Pre-university school	75	17.8
	Diploma/TVET	65	15.4
	Bachelor’s degree	112	26.5
	Master’s Degree and above	28	6.6
Religion	Orthodox Christian	225	53.3
	Muslim	93	22.0
	Protestant	78	18.5
	Catholic	21	5.0
	Others	5	1.2
Occupation	Employed	292	62.2
	Merchant	45	10.7
	Private business	70	16.6
	Others	15	3.6

About 46.7% of the respondents had 1–2 children and more than half (53.6%) reported that they heard about vasectomy. Out of those respondents who heard of vasectomy nearly a quarter (22.3%) obtained the information from their friends. In this study, the knowledge obtained from family planning service providers was reportedly 9.9% [Table 2].

**Table 2:** Reproductive health characteristics and source of information about vasectomy as a method of contraception among married men live in Gulele sub-city, Addis Ababa, Ethiopia 2018 [*n* = 422]

Variables		Frequency (N)	Percent (%)
Number of children	None	109	25.8
	1–2 children	197	46.7
	3–4 children	97	23.0
	5 – above	19	4.5
Have you ever heard about vasectomy?	Yes	226	53.6
	No	196	46.4
Information for availability of vasectomy service	Yes	175	41.5
	No	247	58.5
Where would do you go to seek help?	Hospitals	143	57.7
	Private institution	61	24.7
	H.C	43	17.3
	NGO	1	0.4
Source of information for a vasectomy^a^	Mass media	37	9.9
	HEWs	32	8.6
	Health care worker	75	20.1
	Literature	68	18.2
	Friends	83	22.3
	Through education	78	20. 9

^a^Multiple responses

### Men’s knowledge and attitude towards vasectomy use

The mean knowledge of men towards vasectomy was 5.00 (SD ± 2.66). About 34.8% of the respondents scored greater than the mean score which was categorized as good knowledge. While the mean attitude of married men towards vasectomy was 31.42 (SD± 5.32), about 23.2% of the respondents scored greater than or equal to the mean which was categorized as a positive attitude towards the use of vasectomy.

### Intension to accept or reject vasectomy as a method of family planning

**Fig. 1:**
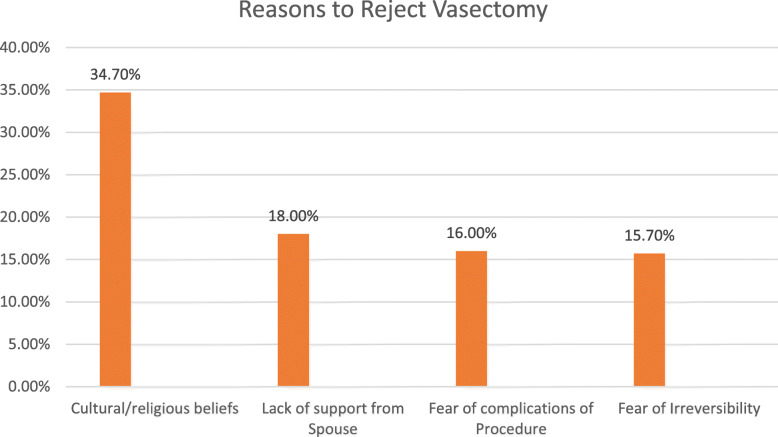
Reasons to accept vasectomy as a method of contraception among married men in Gulele sub-city, Addis Ababa, Ethiopia 2018

**Fig. 2:**
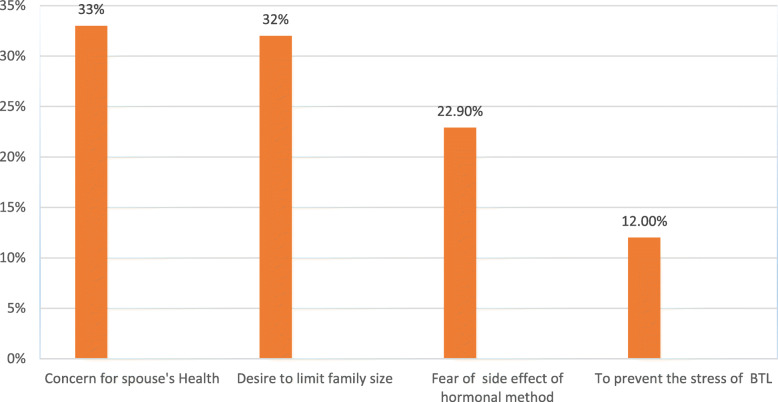
Reasons for rejection of vasectomy as a method of contraception among married men live in Gulele sub-city, Addis Ababa, Ethiopia 2018

More than 3/4th (76.1%) of the respondents wouldn’t accept vasectomy as a family planning method. Their major reasons for refutations of vasectomy as family planning method were included cultural/religious beliefs (34.7%), lack of support from the spouse (18%), fear of complications of the procedure (16%), fear of irreversibility (15.7%) [Fig. 1]. On the other hand, of those reported to accept vasectomy as a family planning method, they reported their main reasons as having a concern for their spouse’s health (33.1%), desire to limit the family size (32%), fear of the side effect of the hormonal method (22.9), and to prevent the stress of bilateral tubal ligation (12%) [Fig. 2].

### Factors associated with intention to use vasectomy

The number of children, religion, and ethnicity were not significant at the bivariate level of analysis. In multivariate analysis, age range between 30 and 39 years [AOR = 2.4, 95% CI = (1.16–4.82)], having a good knowledge about vasectomy use [AOR = 6.22, 95% CI = (3.17–12.21)], and having a positive attitude toward vasectomy use [AOR = 7.81, 95% CI = (4.25–14.38)] were factors significantly associated to use vasectomy as compared to their counterparts [Table 3].

**Table 3:** Factors associated with intention to use vasectomy as a method of contraception among married men in Addis Ababa, Ethiopia 2018 [*n* = 422]

Variables	Intention to Use Vasectomy N (%)		COR (95% CI)	AOR (95%CI)	*P*-Value
	Yes	No			
**Age (in years)**					
20–29	21(20.8)	74(23.1)	1	1	
30–39	56(55.4)	99(30.8)	1.99(1.11,3.58) *	2.36(1.16, 4.82) *	0.018*
40–49	16(15.8)	107(33.3)	0.53(0.26,1.08)	0.83(0.364, 1.89)	0.655
50–59	7(6.9)	26(8.1)	0.95(0.36,2.49)	1.05(0.33, 3.38)	0.932
60–69	1(1.0)	15(4.7)	0.24(0.03,1.88)	0.21(0.02, 2.03)	0.179
**Educational level**					
≤ 10th grade complete	28(27.7)	114(35.5)	1	1	
Pre-university school complete	14(13.9)	61(19.0)	0.93(0.46,1.90)	0.74(0.29, 1.84)	0.512
Diploma/TVET	16(15.8)	49(15.3)	1.33(0.66,2.68)	1.03(0.43, 2.49)	0.945
Bachelor Degree	36(35.6)	76(23.7)	1.93(1.09,3.42) *	0.49(0.20, 1.21)	0.122
Master’s Degree and above	7(6.9)	21(6.5)	1.36(0.53,3.51)	0.31(0.09, 1.08)	0.065
**Occupational status**					
governmental	33(32.7)	91(28.3)	1	1	0.590
non-governmental	41(40.6)	127(39.6)	0.89(0.52,1.52)	0.83(0.43, 1.62)	0.153
merchant	7(6.9)	38(11.8)	0.51(0.20,1.25)	0.42(0.13, 1.38)	0.624
private business	18(17.8)	52(16.2)	0.96(0.49,1.87)	0.80(0.332, 1.94)	0.522
others	2(2.0)	13(4.0)	0.42(0.09,1.98)	0.57(0.99, 3.23)	
**Have you ever heard about vasectomy?**					
Yes	71(70.3)	155(48.3)	2.54(1.57,4.09) *	1.18 (0.44, 3.32)	0.738
No	30(29.7)	166(51.7)	1	1	
**Do you know where vasectomy service is available?**					
Yes	59(58.4)	116(36.1)	0.40(0.26,0.64) *	1.39 (0.58, 3.32)	0.459
No	42(41.6)	205(63.9)	1	1	
**Knowledge**					
Good knowledge	67(66.3)	80(24.9)	5.94(3.66,9.63) *	6.22(3.17,12.21) *	0.00*
Poor knowledge	34(33.7)	241(75.1)	1	1	
**Attitude**					
Positive attitude	65(64.4)	78(24.3)	6.34(3.85,10.46) *	7.81(4.25,14.38) *	0.00*
Negative attitude	36(35.6)	243(75.7)	1	1	

*Significant at ***p*** **< 0.05**

## Discussion

This is a community-based cross-sectional study that investigated the factors that determine the intention to use vasectomy among 422 married men in the Gulale sub-city of Addis Ababa, Ethiopia. Reproductive health decision-making is the shared responsibility of men and women. One of the most important indicators of reproductive health is the effective utilization of family planning [8]. Most FP methods often focus solely on women, with the objectives of preventing recurrent births and reducing maternal and fetal death. While vasectomy is an easy procedure with a high achievement rate (> 99%) and minimum complications including swelling and pain, it is yet underutilized across the world, mostly in developing nations [13, 14, 17]. The global rate of vasectomy use is stated at 3%, with a rate of 2% in developed countries [18]. Canada (22%), China (21%), the United Kingdom (21%), South Korea (16.8%), the United States (12.7%), and Australia (9.3%) have the best utilization rates. On the contrary, developing countries such as India (0.1%), the Philippines (0.1%), Ghana (0.0%), and Cuba (0.1) have a totally low rate of vasectomy use [18, 19].

According to the Ethiopian Demographic Health Survey 2016 report, the rate of vasectomy use is very low in Ethiopia (< 1%) [2]. Determining the intention to use vasectomy as a method of contraception is hence an important indicator of the potential demand for Family Planning services. Understanding the characteristics of married men to use vasectomy might offer awareness to demand future use of long-acting permanent methods of family planning (LAPMs).

In this study, the intention to use vasectomy by the study subjects was only 24% (95% CI = 34.1, 42.7). However, the level of acceptance of vasectomy (24%) was high compared to the level of its use in developing countries. It is higher than Canada (22%); that is if acceptability translates to use. In our present study, more than 3/4 (76%) of the study subjects detailed that they could never utilize vasectomy as they perceived that it hurt their marriage and sexual wellbeing, which is steady with the study conducted by Kisa and partners on the perspectives of wedded couples living in Turkey towards vasectomy, in which it was accounted for that 88% of wedded men demonstrated hesitance to acknowledge vasectomy as a family planning strategy [17]. Notwithstanding, our finding is lower than the surveys conducted in Nigeria and Nepal [15, 16].

The current study provides some plausible reasons for refusing to adopt vasectomies such as cultural/religious beliefs, lack of support from a spouse, fear of complications of the procedure, fear of irreversibility, and fear of impotence among others. This finding is consistent with studies conducted in East Wollega, Ethiopia, Nigeria, and central India [20–22]. Kisa and partners stated comparable findings from Turkey where socio-social elements (counting convictions) that contraception is the lady’s obligation, and that vasectomized men may lose status in the public eye and authority in the family were the primary hindrances for vasectomy use in Ethiopian culture and somewhere else because of fears related to sexual relations, mental impacts, and consequences for physical quality, and so on [17].

Culture and community aspects impact the willingness of men to use vasectomy as a contraceptive method in Africa including Ethiopia where male-dominancy is prevalent. The use of vasectomy is supposed to be discouraged by fears of castration, loss of erectile function, loss of libido, and sociocultural factors such as the risk of sexual disability after vasectomy and a sense of degradation [14, 19, 20].

The present study showed that 2/3rd (65.2%) of the participants had poor knowledge about vasectomy use as a method of contraception, and more than 3 /4th (76.8%) of the respondents had a negative attitude towards intention to use vasectomy. On the other hand, knowing vasectomy use, having a positive attitude about vasectomy use, and the age of men had shown significant association with the use of vasectomy as a contraceptive method, which was comparable with studies conducted in Western Nepal and east Wollega, Ethiopia [16, 20]. This comparability might be due to the similar socio-economic, socio-cultural, and educational status of the participants. Nonetheless, this finding is less than the study finding from India [16]. This difference might be health care providers might not advocate vasectomy service as a method of family planning in Ethiopia as the knowledge obtained about vasectomy use from providers was reportedly 9.9% in this study. Thus, media (including state media) should be considered as an important channel to reach married couples with no prior information about Reproductive health information including, vasectomy use. The government should show concerns regarding technology as a means to deliver health information and interventions on the family planning program, which encompasses male participation (in this case vasectomy use).

Having a positive attitude toward vasectomy use had shown a significant association with the use of vasectomy as a contraceptive method in our study, which was consistent with studies conducted in Western Nepal (60%) and in Nigeria (62.7%) where the study participants had a positive attitude towards vasectomy [16, 19]. However, this finding was not correlated with a study conducted in Nigeria where 82% of the participants had a negative attitude towards vasectomy [21]. This difference could be due to the difference in health information, educational background, and the difference in health coverage.

Another key finding of this study was that participants’ educational qualifications showed no association with their level of knowledge about vasectomy use, which was consistent with the previous study from Nigeria [23].

### Limitations

This population-based study was the first to assess the intention to use vasectomy as a method of contraception among married men in Ethiopia. However, the findings of this study could only be generalized to this cohort of married men in the study setting. The study would have benefited by conducting the study among both married men with their spouses to compare their acceptability and intention to use contraceptives. However, this study involved only married men and not their spouses, which might raise a research question: Will women choose vasectomy as a method of contraceptive for their partners? This is a potential area for future researchers. As in all cross-sectional studies, we can infer association but not causation.

## Conclusions

The level of acceptance of vasectomy (24%) was high compared to the level of its use in developing countries (i.e. if acceptability translates to use). Knowledge of vasectomy, having a positive attitude about vasectomy use, and the age of men had a significant association with the use of vasectomy as a contraceptive method. The study provides some plausible reasons for refusing to adopt vasectomy such as lack of support from a spouse, fear of side effects among others. Nevertheless, the authors didn’t explore the issue of quality in the provision of vasectomy to potential family planning users. Likely, some married men would not use vasectomy because they did not know where to obtain the service, or they were not aware of the correct potential side effects and how to manage the side effects should they occur. Hence, to further promote the use of vasectomy effective communication strategies in family planning programs are crucial. The study also recommends designing strategies to overcome sociocultural barriers by raising awareness to surge vasectomy use. Couple-specific family planning programs may contribute toward enhancing awareness of the benefits of vasectomy. Further qualitative study is also required to better understand the perspectives of married couples towards the use of vasectomy as a method of family planning. The issue of quality in the provision of vasectomy to potential family planning users is another prospective research area.

## Data Availability

The datasets used and/or analysed during the current study available from the corresponding author on reasonable request.

## Abbreviations

FP: Family planning
EDHS: Demographic Health Survey
TFR: Total fertility rate
LAPMs: Long-acting and permanent methods
IUD: Intrauterine device
